# A method for testing health system resilience: Development, application and lessons learned^☆^

**DOI:** 10.1016/j.healthpol.2026.105618

**Published:** 2026-06

**Authors:** Julia Zimmermann, Philip Haywood, Marina Karanikolos, Jonathan Cylus

**Affiliations:** a European Observatory on Health Systems and Policies, London School of Economics and Political Science, London, UK; b University of Sydney, Sydney, Australia; c European Observatory on Health Systems and Policies, London School of Hygiene and Tropical Medicine, London, UK

**Keywords:** Resilience, Health system resilience, Health system resilience testing, Preparedness planning, Health system performance assessment, Health security

## Abstract

**Background:**

Recent adverse events, such as the COVID-19 pandemic, economic crises, conflict, migration and extreme weather have demonstrated how a wide range of shocks can challenge health systems. While emergency preparedness and planning is a well established component of health system governance in many countries, no methodology has existed to systematically test the resilience of a health system to a broad range of shocks.

**Objective:**

To share lessons learned from conducing resilience testing exercises.

**Methods:**

The resilience testing methodology is a mixed-method country-led exercise designed for policy making. We developed this method to test the resilience of a health system. A specific shock scenario is chosen from a broad range of possible shocks to hypothetically challenge a given health system to its limits. The impact of the shock is assessed using the global HSPA framework and the shock cycle framework. The process culminates in a semi-structured resilience testing workshop that brings together policy makers, civil servants and other relevant stakeholders. The workshop is designed to identify key structural strengths and weaknesses of the health system, which then can be improved through remedial policy action. In this paper we use the RE-AIM framework to present findings from our mixed-methods formative evaluation.

**Results:**

Successful approaches involved carefully selected facilitators, a diverse range of participants performing different health system functions and support from the Ministry of Health.

**Conclusions:**

Resilience testing can identify health system strengths, weaknesses and opportunities for remedial policy action.

## Highlights

• Health system resilience testing is a country-led exercise for policy making
• Resilience testing can accommodate a broad range of shocks and country contexts
• Resilience tests identify strengths, weaknesses and opportunities for policy action
• Support from the authorities is important for successful resilience testing

## Background

Health system resilience has gained traction in the last two decades following a series of disasters that have affected the global community [1]. The COVID-19 pandemic demonstrated that resilience is a property of health systems that determines how well this system fares while experiencing a shock, with some health systems mounting a more resilient response than others [2]. In an environment marked by permacrisis efforts continue to understand which attributes of a health system contribute to its resilience [[3], [4], [5], [6]].

Health system resilience has many definitions and different approaches to operationalization [7]. Many of these approaches have been summative and retrospective, such as the studies that have evaluated the response of a health system facing a shock [[8], [9], [10]]. Following a recommendation from the EU Expert Panel on effective ways of investing in health on the organization of resilient health and social care following the COVID-19 pandemic [11], the European Commission tasked the European Observatory on Health Systems and Policies and the OECD to develop a methodology to test the state of health system resilience in Member States with the view of detecting weaknesses and finding policy solutions for remedial action, to improve responses to future shocks. Accordingly, we developed a new methodology for resilience testing in association with national and regional governments in Europe [12]. In this paper, we share learning from the formative project evaluation, aiming to share learnings on which approaches help deliver successful resilience testing.

## Methods

### Description of health system resilience testing

The *Strengthening Health Systems: A Practical Handbook for Resilience Testing,* provides step by step instructions and worked examples to conduct a resilience test [12]. It describes how to prepare for, conduct and follow up a resilience test. The process requires a small team of resilience test organizers and takes 4-6 months, from initiation to completion.

The resilience test is anchored in a hypothetical shock scenario. The choice of scenario is very flexible and can describe many different types of shock, from a pandemic to a natural disaster, to a political crisis etc. Regardless of the chosen scenario, the shock needs to push the health system to breaking point so that the strengths of the health system and the weaknesses that hinder a resilient response are highlighted.

Resilience testing uses a mixed-methods approach underpinned by the global HSPA framework and the shock cycle framework [13,14]. Once the scenario has been determined, existing health system data is collected and analyzed. This background research serves to inform discussions on the resilience test day. The data collection draws on existing national and international indicators and needs to be tailored both to the shock scenario and to the data available within the health system. The WHO has recently released a compendium of health system resilience indicators [15], which may be used to supplement example indicators that are described by the Handbook.

The resilience test day is a semi-structured workshop that brings together relevant stakeholders. Discussions aim to understand the resilience of the health system if it were faced with the hypothetical shock scenario. Participants vote on the perceived strengths and weaknesses of the health system, given the scenario, throughout the test day. The voting results are supplemented by notes from the day’s discussions. The information from the background research, notes and voting results inform the resilience test’s follow up.

### Evaluation of health system resilience testing

The resilience testing methodology was developed iteratively between January 2022 and March 2024. The steps included a literature review of resilience testing. Econlit and Pubmed databases were searched in January 2022 using the following search string: "Public health policies“ & “Health systems resilience” & ("International" or "Countries" or "country level analysis" or "cross-country" or "worldwide" or "health system characteristics"). This search was supplemented by snowball searching and by current work from the OECD, European Observatory on Health Systems and Policies and the European Commission. A review of the appropriateness of different shock cycles and HSPA frameworks was conducted with experts within the OECD, European Commission, ECDC and WHO, and refinement was achieved using specific examples and presenting them to expert panels before resilience tests were conducted. Resilience tests were conducted in Finland, using a pandemic scenario that primarily affects children (April 2023), Greece, using a scenario describing a large exodus of health workforce (May 2023), and in the Region of Asturias, Spain, using a heatwave scenario (November 2023). Resilience tests followed the methodology outlined in the Handbook [12] and were prepared and facilitated by a team of local resilience tests organizers. The resilience testing project team from the European Observatory and OECD oversaw the preparation for each resilience test and were joined by a representative from the European Commission to observe the proceedings on each resilience test day.

We conducted a mixed-methods evaluation of each resilience test aimed at enhancing the useability of the new methodology. We took a formative approach and used the results to iterate on the resilience testing methodology between tests. We collated records of resilience test day preparations, observed the resilience test days and conducted an online questionnaire that resilience test participants were asked to fill out at the end of the test day. The questionnaire was developed using two rounds of internal feedback and was tested with an independent researcher. Questions that used a Likert-scale were converted to a numeric value for analysis where 5=strongly agree, 4=agree 3=neutral, 2=disagree, 1=strongly disagree. Notes were taken by at least 2 observers for each resilience test. Observers filled out a pre-prepared note taking pro-forma. We also conducted a semi-structured interview with resilience test facilitators and organizers the day after the resilience test workshop. The semi-structured interview topic guide was also developed through two rounds of internal feedback and also tested with an independent researcher. The same person conducted the interviews each time. Interviews were not recorded and notes were taken to capture responses during the interviews. Please find the questionnaire, semi-structured interview topic guide and note-taking proforma for resilience test observations in the supplementary materials. We present overarching findings using elements of the RE-AIM framework, which is a frequently used implementation framework ([16,17]). We focused on “Reach”, which analyses participation, “Effectiveness”, which analyses whether the resilience test was achieved, and “Adoption”, which analyses factors important in the initiation of resilience testing.

## Results

### Reach

After consent from the national authorities to conduct the resilience test had been obtained, resilience test organizers were recruited. Resilience test organizers were experts in the national health system and seen as independent and politically neutral. All facilitators had prior facilitation experience. A team of five experts organized and facilitated the Finnish resilience test. Three experts organized and facilitated the Greek resilience test and four experts organized and facilitated the resilience test in Asturias.

Semi-structured interviews with facilitators showed that the perceived independence and neutrality of organizers and facilitators were seen as key to a successful resilience test. Good knowledge of the health system, health system stakeholders and subject-matter expertise related to the scenario were also seen as helpful. All facilitators thought that using two resilience test facilitators on the test day helped create a dynamic and focused discussion, while two additional team members ensured comprehensive note-taking and smooth logistics.

Test organizers and facilitators also discussed the right number and composition of participants. A group of 15 participants was deemed ideal, with resilience tests up to a maximum of 25 participants possible, to allow for balanced discussions given time constraints. It was agreed that the categories of stakeholders should be identified by the test organizers, rather than the ministry of health, by means of a stakeholder analysis. They also reflected on the value of a wide range of participants, including frontline health care workers, such as doctors, nurses and allied health professionals, representatives from relevant ministries and a representative from the ministry of health.

The resilience tests had 18 (Finland), 13 (Greece) and 13 (Asturias) workshop participants (excluding researchers and facilitators). Participants thought they had sufficient opportunities to contribute to the exercise, scoring a mean of 4.8 on the 5-point Likert scale. Descriptive statistics of the main participant questionnaire findings are presented in the supplementary material.

27 experts were invited to the Finnish resilience test and 19 experts to resilience tests in Greece and Asturias. Therefore, the attendance ratio was 67% for the Finnish test and 68% for the other two tests. All three resilience tests succeeded in attracting high-level participants. A high-level politician attended and contributed to one of the resilience tests. The other two resilience tests were intentionally conducted without the involvement of politicians. When asked to self-assess whether participants had sufficient knowledge of the health system to constructively contribute to the discussions, the mean score across all resilience tests was 4.3 out of 5 on a 5 point Likert scale.

In the online survey results, responses to the question “Are there any types of participants who did not attend the Health Systems Resilience Test who you think could have usefully participated?” were varied. Most responses, that specified additional participants, identified representatives from functions in sectors beyond the health sector (such as education or transport) that were relevant to the shock scenario. Some participants identified representatives from health system sub-functions that were not present on the day and one participant suggested including more diverse participant profiles.

In all three tests, the resilience test organizers and facilitators received mostly positive feedback on resilience test invitations. Irrespective of the notice period, some invitees were limited by conflicting priorities. Very few invited participants did not respond to the resilience test invitation, and some invitees sent a deputy to represent their health system function. Test organizers reported that a request for engagement from the authorities and existing institutional relationships were important to ensure response and participation. The presence of international organizations as observers was seen as a pull factor, reinforcing the priority and legitimacy of the event.

### Effectiveness

#### Resilience test day preparations

Most participants thought that the pre-test information had been suitable preparation for them. Across the three tests, the mean score of whether the purpose and objectives of health system resilience had been clearly communicated in advance was 4.3 on the 5-point Likert scale. The mean score for whether the background materials had been clear was 4.5. The mean score for whether the background information had prepared participants for the resilience test day was 4.2. Two participants commented that the objective of the resilience test had not been made clear. One participant would have liked to see international comparisons and another participant would have liked to see more figures as part of the background materials. For one of the tests, the background information had only been sent out the day before, and three participants from this test commented that they would have liked to have receive the background materials earlier.

In the post-test interview, resilience test organizers and facilitators reflected on the amount of data needed to prepare for a resilience test. While they wanted to provide sufficient data to inform discussions, it was not deemed helpful to provide too much data, especially as participants were chosen for their existing expertise. Challenges included lack of data, gaining access to existing data and compiling data kept by different institutions. International comparisons were deemed less important than national data. We learned that it is helpful to have more detail on data that is potentially controversial, including sources and methods, so that these can be explained during the resilience test if challenged.

In the post-test interview, resilience test organizers also reflected on the extent to which they should share details of the testing methodology with participants in advance of the test day. On the one hand, participants should be informed and feel prepared. On the other hand, the resilience test should not be seen as an academic exercise or an intimidating evaluation. All resilience tests included a presentation summarizing the resilience testing process at the beginning of the test day. Also, there was a suggestion that future resilience test organizers could include the objectives of the resilience test in the background materials sent to participants.

#### Resilience test day proceedings

Overall, participants thought that the resilience test day was well conducted and facilitated. In all three tests, the facilitation of the resilience tests scored a mean of 4.6 on a 5-point Likert scale. Participants felt empowered to contribute (4.5) and the visual tools used during the facilitation were deemed helpful (4.5). Nine participants left positive general comments and described the resilience test day as well-run, practical and an overall good experience. Critical general comments included a suggestion to reveal the scenario in stages rather than all at once, that there had been too many votes on the test day and one participant questioned whether the resilience testing method is suitable for health system evaluation.

Facilitators and test organizers reflected on the facilitation. It was agreed that it was important to engage the participants and create an environment conducive for a constructive discussion through an icebreaker exercise at the beginning, such as the initial voting exercise. It was also important that facilitators remained neutral and refrained from controlling the narrative, but that they challenge participants’ statements or provide contextual information where appropriate. Facilitators suggested that future resilience test facilitators should build their awareness of existing power dynamics between participants in preparation for the resilience test. During the test with a high-level political participant, and thus an imbalance of power, the participant was asked to speak last and allow all others to contribute first. This was a successful strategy that encouraged open and self-reflective discourse during the resilience test. Other strategies identified as helpful were ensuring that the logistics of the resilience test day were appropriately set up, including details such as the layout of the room, name placards and sufficient microphones. It was also deemed important to have sufficient breaks during the day. There was discussion around the role for “external observers” of the resilience test. Resilience test organizers and facilitators thought that participants took the resilience test more seriously because international health system experts were in the room. They also thought that discussion dynamics could have potentially been easier to manage because of this presence.

#### Resilience test day outputs

All resilience tests successfully identified key health system strengths and weaknesses in relation to the shock scenario. Test organizers and facilitators reported that the assessment of resilience during the test day as well as discussions accurately reflected the state of the health system. They thought that participants raised a few practical options for remedial action that could be implemented easily and within a good timeframe.

Participants thought that the test outcomes were an accurate reflection of the conversations during the resilience test day, scoring a mean of 4.2 on a 5-point Likert scale. In most cases the results were consistent with the participant’s expectations of the major weaknesses of the health system and only one participant did not expect the results.

All resilience tests used interactive online tools to allow participants to vote to evaluate the resilience of their health system. Facilitators had received verbal feedback from participants that they enjoyed this part of the resilience test day, especially in contexts where such tools are not in common use. Initially, discussion and voting were conceptualized per health system function, and the first resilience test demonstrated that discussing and voting per stage of the shock cycle flowed better. Subsequent tests took this further to include 10-15 summarized points that had come up during the discussion in the vote and maintained a pre-set list of criteria for the first and last vote. Facilitators noted that this helped the participants feel involved in shaping the discussion, but also led to some push-back where more contentious points that had been raised were reflected back to participants.

### Adoption

#### Time taken

Most participants spent around 30 minutes reviewing the background material that had been sent out prior to the resilience test day (range 5min-3h). The resilience test itself required one full working day of participant time. All but one participant across the three resilience tests agreed that a full working day was sufficient time to run through the exercise.

Resilience test organizers and facilitators spent between 4 and 8 weeks preparing for the resilience test. There was some discussion on whether a single day was sufficient to cover the resilience test, as each resilience test had a lot of material to be prioritized and integrated. There was agreement that participants would be unlikely to be able to attend two consecutive days. However, a follow up resilience test a few weeks or months after the initial resilience test was deemed more realistic. More time would allow for further thinking about the weaknesses that had been identified, and to discuss remedial action in more detail. More time could also allow the resilience test organizers to replace voting with a qualitative process to identify priorities.

### *Ease of using the resilience testing methodology*

All resilience test organizers conducted a successful resilience test using the handbook for resilience testing and 3-5 preparatory meetings with the resilience testing project team. They reported that they initially found the distinction between health system performance assessment and assessing health system resilience difficult to grasp. The examples that were provided and the discussions with the project team were deemed helpful for understanding this distinction and clarifying the intended process. There was also some initial confusion when using the conceptual framework for resilience testing as in some scenarios not all parts of the framework are applicable.

Participants in the first resilience test sought to clarify the difference between a resilience test and other scenario-based preparedness exercises. In subsequent resilience tests, this explanation was included in the presentation of the methodology and the same queries did not recur.

Some participants were very critical of the shock scenarios used in two of the resilience tests. These participants pointed out scenario deficiencies during the resilience test day. In the participant questionnaire, six participants commented on scenario deficiencies. The overall relevance and appropriateness of the shock scenario scored mean of 4.0 on a 5-point Likert scale.

Resilience test organizers and facilitators confirmed that all shock scenarios had been reviewed by experts in the field. They agreed that expert review of scenarios was essential, both to ensure that the scenario is as realistic as possible, and to confirm this to resilience test participants should the scenario be questioned. Test organizers and facilitators also advised that future test organizers should prepare the shock scenario as early as possible, as the scenario helps to determine the stakeholders who would be relevant to the resilience test.

#### Value of resilience testing

Most participants found the resilience test a valuable exercise with a mean score of 4.4 on the 5-point Likert scale. All but one participant agreed that the resilience test also helped identify broad health system vulnerabilities (not specific to the shock scenario). In two of the tests, 100% (13/13) and 83% (10/12) participants thought that they would have identified different health system strengths and weaknesses had they used a different shock scenario. 33% (3/9) of participants agreed with this statement in the third test. The resilience test contributed positively to participants’ thinking on health system resilience. Five participants commented that the test gave them a better understanding of the health system. Two participants commented that it provided an opportunity to reflect, and two participants commented that the test enabled them to hear different points of view. One participant remarked that the test had made them aware of the strategic importance of health system resilience.

The facilitators and resilience test organizers had received positive verbal feedback from participants, who had appreciated the chance to share aspects of their work with a group of stakeholders representing different health system functions. Many participants also found the conceptual frameworks underpinning the resilience test helpful to better understand their health system.

Facilitators agreed that some vulnerabilities that were discussed would apply across shock scenarios e.g. identifying the vulnerable population. However, most of the discussions were very specific to the shock scenario and may not necessarily be relevant in other shock scenarios. Overall, facilitators felt empowered to objectively evaluate the health system in the context of the chosen shock scenario.

## Discussion

The resilience tests identified health system strengths and weaknesses related to a hypothetical shock scenario. Most participants thought that the resilience test day was useful, provided them with opportunities to discuss their work from different perspectives and contributed to their understanding of the health system. Moreover, resilience testing was intentionally designed to be achievable by a small team within the timeframe of less than half a year. This demonstrates that health system resilience testing is feasible, able to assess health system resilience and identify areas for potential remedial action.

There is no uniform template for assessing resilience as health systems and their interactions with shocks are varied and complex. Nonetheless, we found that successful approaches involved a few core ingredients. Firstly, the resilience test facilitators need to be carefully selected. Facilitators need to be experienced at managing group discussions, experts in the national health system and seen as independent from the government. Secondly, resilience testing benefits from a diverse range of participants who can speak for various functions in and beyond the health system. It is a careful consideration to ensure a broad and balanced representation while maintaining a group size conducive to discussion. Lastly, endorsement from the Ministry of Health not only ties resilience testing into the policy making process, but also helps prioritize scenario choice and provides convening power.

The resilience literature has grown immensely in the last few years and various approaches to understanding health system resilience with the view of identifying opportunities to improve it have been developed. Although we only became aware of the work of Ebi et al. [18] after publication of our Handbook for Resilience testing, they describe a reassuringly similar approach that uses scenarios, desk research and stakeholder involvement, albeit centered on improving climate resilience rather than health system resilience. In the recent health system resilience literature, system dynamic modelling and realist review techniques have been used to identify opportunities to improve health system resilience. Ager and colleagues [19] describe how they used system dynamic modelling in the context of a low and middle income country to identify levers to improve health system resilience. Loffreda and colleagues [20] have used similar methods to understand the health system’s interaction with other systems in the context of climate change. Fleming and colleagues [21] use realist review techniques as a means of identifying causal pathways and opportunities for change, focusing on the health workforce. In all cases, as in the case of our resilience testing method, stakeholder engagement was used to confirm, refute and refine emerging results on how the system works and what could be done to improve it. It appears that stakeholder engagement could be a crucial component of any exercise that aims to improve health system resilience.

As our primary aim was neither to conduct a formal evaluation, nor a qualitative research exercise, our methods were restricted by time with a limited group of people. Therefore, we are drawing conclusions based on our best interpretation of the data we have collected. Due to the large increase in resilience literature following the COVID-19 pandemic, the results of the literature review would be likely to change if it were run at a later date. The professional background of all facilitators, test organisers and participants of the resilience tests were representative of the participants we would expect to fulfill these roles in future resilience tests and most results were consistent across all three tests, indicating that collecting further data would have been unlikely to change our overall findings. While we are not able to attribute responses to different professional backgrounds, as this may identify participants, there is scope for future research to examine the role and responses of stakeholders from different professional backgrounds in resilience testing.

The main limitation of resilience testing is that it relies on a hypothetical shock scenario rather than an actual shock, which limits the ability to detect unanticipated outcomes [22]. Resilience testing is also designed to be a tool for policy making, and outcomes derived from the participatory exercise may not have academic rigor. For example, the selection of test organizers, facilitators and participants may impact upon resilience test outcomes and how well the outcomes represent the health system’s function during an actual shock. While resilience testing can detect weaknesses in health system resilience that are relevant to other scenarios, many of the discussions may remain specific to the shock scenario. It therefore would be necessary to run several different scenarios to build an overview of health system resilience to multiple shocks. The evaluation used some but not all of the elements of the RE-AIM framework, the longer term implications of resilience testing in altering health systems were not assessed.

The testing and interaction taught several important lessons that were reflected in the Handbook. Future research could allow for data collection on implementation and maintenance and formal evaluation, which would improve the robustness of evidence. Maintenance of resilience testing will largely depend on government buy-in. We found that government consent is critical, and all resilience tests were government led in the first place. This facilitates bringing resilience test participants to the discussion and ties resilience testing results into the policy making process. This mirrors findings from policy dialogues, where support from empowered public agencies is a critical condition [23]. The governments we have worked with found the resilience tests useful and to our knowledge, two of the three tests have since contributed to policy making. Outside of the support the European Commission provided to develop this methodology, it is now up to national governments to take up resilience testing. For example, resilience testing could become an institutionalized governance process to regularly assess the health system’s resilience.

## Conclusion

Resilience tests can identify health system strengths and weaknesses, and steps towards remedial policy action to improve health system resilience at national and regional level in different European contexts. Results may depend on scenario selection, and several resilience tests using different scenarios may need to be conducted for a full view of health system resilience. We found that successful approaches involved carefully selected facilitators, a diverse range of participants performing different functions and support from the Ministry of Health.

The benefit of resilience testing may go beyond identifying health system strengths, weaknesses and opportunities for remedial action, to include the ability to bring together stakeholders from across the health system and relevant sectors, thus breaking down established silos. Ultimately, the value of resilience testing will be the impetus it may provide for evidence-based reform of health systems, so that they are better equipped to withstand current challenges and future shocks.

## Research in context

1. **What is already known about the topic?** Health system resilience has many definitions and different approaches to operationalization.
2. **What does this study add to the literature?** We developed and evaluated a methodology to test the resilience of a health system against a broad range of shocks. We learned that successful resilience testing requires careful selection of facilitators and participants as well as support from the Ministry of Health.
3. **What are the policy implications?** Resilience tests are designed for policy making and can identify health system strengths, weaknesses and steps towards remedial policy action to improve health system resilience at national and regional level in different European contexts.

## Funding

The resilience testing project was funded by the European Commission (Call EU4H-2021-DGA-IO2-IBA, Project number: 101070819).

## CRediT authorship contribution statement

**Julia Zimmermann:** Writing – review & editing, Writing – original draft, Methodology, Investigation, Formal analysis, Data curation, Conceptualization. **Philip Haywood:** Writing – review & editing, Methodology, Investigation, Conceptualization. **Marina Karanikolos:** Writing – review & editing, Methodology, Conceptualization. **Jonathan Cylus:** Writing – review & editing, Supervision, Methodology, Conceptualization.

## Declaration of competing interest

None.

## Appendix Supplementary materials

Image, application 1
