# Supplementary material for: A method for testing health system resilience: Development, application and lessons learned
Source: Health Policy. 2026 Jun;168:105618. doi: 10.1016/j.healthpol.2026.105618 (PMC13082236; doi:10.1016/j.healthpol.2026.105618)
Supplement: Supplementary file 1 [file mmc1.docx]

# Supplementary Material: Participant Feedback Questionnaire

This feedback form will be used to gather feedback on the Health Systems Resilience Testing Pilot and to inform adjustments to the approach and methodology. These questions should be answered by individuals who participated in the Health Systems Resilience Test.

## Preparation and Communications

1. Were the purpose and objectives of the Health Systems Resilience Test clearly communicated to you in advance? Please select one box.

|  |  |  |  |  |
| --- | --- | --- | --- | --- |
| 1 Not at All | 2 | 3 | 4 | 5 Very Clear |

2. Were the background materials sent to you in advance clear and easy to understand? Please select one box.

|  |  |  |  |  |
| --- | --- | --- | --- | --- |
| 1 Not at All | 2 | 3 | 4 | 5 Very Clear |

3. Did the background materials sent to you in advance prepare you to participate in the Health Systems Resilience Test? Please select one box.

|  |  |  |  |  |
| --- | --- | --- | --- | --- |
| 1 Not at All | 2 | 3 | 4 | 5 Very Well |

4. Was the shock scenario appropriate and relevant? Please select one box.

|  |  |  |  |  |
| --- | --- | --- | --- | --- |
| 1 Not relevant | 2 | 3 | 4 | 5 Very relevant |

5. How much time did you spend looking at materials/preparing for the pilot before the test day?

|  |
| --- |

6. Please provide any general comments on the **communications and materials** that you received in advance of the Health Systems Resilience Test.

|  |
| --- |

## Delivery

7. Was the Health Systems Resilience Test facilitated in a clear and logical manner? Please select one box.

|  |  |  |  |  |
| --- | --- | --- | --- | --- |
| 1 Not logical | 2 | 3 | 4 | 5 Very logical |

8: Did you feel empowered to contribute to the exercise?

|  |  |  |  |  |
| --- | --- | --- | --- | --- |
| 1 Did not feel empowered | 2 | 3 | 4 | 5 Felt very empowered |

9. Did you have sufficient time to run through the exercise and consider the resilience of your health system in response to the shock scenario? Please select one box.

|  |  |
| --- | --- |
| Yes | No |

10. Did you have sufficient opportunities to contribute to the exercise? Please select one box.

|  |  |  |  |  |
| --- | --- | --- | --- | --- |
| 1 No opportunities | 2 | 3 | 4 | 5 Sufficient opportunities |

11. Were the visual tools used during facilitation useful in helping you to consider the resilience of your health system? Please select one box.

|  |  |  |  |  |
| --- | --- | --- | --- | --- |
| 1 Not at all | 2 | 3 | 4 | 5 Very useful |

12. Did you have sufficient knowledge about your health system to participate in and contribute to the Health Systems Resilience Test? Please select one box.

|  |  |  |  |  |
| --- | --- | --- | --- | --- |
| 1 Not enough knowledge | 2 | 3 | 4 | 5 Enough knowledge |

## Health Systems Resilience Testing Outcomes

13. Were the test results an accurate reflection of the conversation during the Health Systems Resilience Test? Please select one box.

|  |  |  |  |  |
| --- | --- | --- | --- | --- |
| 1 Not accurate | 2 | 3 | 4 | 5 Very accurate |

14. Where the results of the day consistent with your expectations of the major weaknesses of the health system?

|  |  |
| --- | --- |
| Yes | No |

15. Was the Health Systems Resilience Test Pilot a valuable exercise for you? Please select one box.

|  |  |  |  |  |
| --- | --- | --- | --- | --- |
| 1 Not valuable | 2 | 3 | 4 | 5 Very valuable |

16. Do you think that results from the Health Systems Resilience Test will help you to identify and understand broader vulnerabilities in your health system (not specific to the shock scenario)?

|  |  |
| --- | --- |
| Yes | No |

17. Do you think you or the participants as a group might identify different strengths and weaknesses in the resilience of your health system if you tested it with a different shock scenario?

|  |  |
| --- | --- |
| Yes | No |

18. Do you think the participants have identified reasonable next steps to improve health system resilience to this shock? (Resilience test 3 only)

|  |  |  |  |  |
| --- | --- | --- | --- | --- |
| 1 Not reasonable | 2 | 3 | 4 | 5 Very reasonable |

19. Are there any types of participants who did not attend the Health Systems Resilience Test who you think could have usefully participated?

|  |
| --- |

20. Please provide any comments on how the Health Systems Resilience Test has contributed to your thinking on Health Systems Resilience

|  |
| --- |

22. What do you expect the next steps after the resilience test will be? (Resilience test 3 only)

21. Please provide any general comments on the Health Systems Resilience Test and suggestions for improvement

|  |
| --- |

Would you wish to be involved in the process going forward? (Resilience test 3 only)

|  |  |
| --- | --- |
| Yes | No |

# Semi- structured interview topic guide

| 1. **Input** | |
| --- | --- |
| Methodology | Q1. In your opinion, is the pilot test methodology structured in a clear manner?  → follow-up:   - What parts were straightforward and what were not to you? - What do you think can be improved? |
| Preparation on the facilitator's end  (Resources and costs, context) | Q2. How did your preparation for the pilot as a facilitator go?  → follow-up:   - Were there any difficulties? How did you deal with them? - What do you think can be improved? - How much time did you spend on preparing to facilitate the test? - What went well? |
| Support by OECD/OBS  (Information, people, context  ) | Q3. Did OECD/OBS team provide sufficient clarifications, feedback and support during the preparation phase?  → follow-up:   - How helpful was the draft manual? - Did they provide you with sufficient information to prepare the test? |
| 1. **Activities** | |
| Environment | Q1. Was the physical environment suitable for your facilitation of the test (e.g., venue, location, size, set-up, etc.)?  Q2. Was there sufficient time to run through the whole exercise?  → follow-up:   - How long do you think is appropriate for a test duration? |
| Engagement | Q3. In your view, did all participants have sufficient knowledge of the health system in order to contribute to the discussion? |
| Shock cycle/HSPA/  Scenario (Advancing the discussion) | Q4. Was the methodology appropriate to facilitate the discussion?  Q5. What helped/hindered your facilitation? |
| 1. **Output** | |
| Results validity | Q1. Do you think that the results reflect the discussion accurately?  → follow-up:   - Are the results drawn from reasonable justifications? |
| Gaps between the scenario intention and the discussion | Q1. Were the discussed topics what the scenario intended to introduce?  Q2. Did the topics deemed important by participants reflect the scenario's intention to highlight them?  → follow-up:   - If not, why were the topics deemed important? |
| Identifying strengths/  weaknesses | Q1. What lessons were learned about your country's health system through the test?  Q2. Do you think that the test succeeded to spot the health system's strengths and weaknesses accurately?  → follow-up:   - What were the difficulties in identifying them? |
| 1. **Outcome** | |
| Value | Q1. Was the test a valuable exercise to understand the resilience of your country's health system?  Q2. Would you learn another test with a different shock scenario?  → follow-up:   - Why? |
|  | Q1. Was the test scenario with one shock and test duration of one day sufficient to assess your country's health system?  → follow-up:   - Would have it been helpful if multiple shocks or/and more time were given? |
| Applicability | Q1. Do you think that the test outcomes can be applied to understand your country's health system in general (not specific to the shock scenario situation)? |
| 1. **Impact** | |
|  | Q1. In your opinion, would the test results lead to any policy changes?  Q2. What impacts do you think does the test have on your country's health system? |

# Assessment template for the pilot day

## Part 1. Assessment of the pilot

The first part of this assessment template aims to evaluate the running of the pilot, focusing on whether the pilot unfolded within the planned framework with the facilitator and participants fulfilling their tasks. It contains 26 questions divided into 5 sections, each assessing a different aspect of the course of the pilot:

1. Coverage of assessment items
2. Discussion flow and methodology
3. Description and involvement of the participants
4. Role of the facilitator
5. Overall evaluation

| 1. **Coverage of assessment items** |
| --- |
| Q1. Which of the HSPA functions and shock stages did the discussion cover? |
| (1) Yes / Yes, but insufficiently / No + (2) deemed important / deemed unimportant  (e.g., No + deemed unimportant)   \|  \| Preparedness \| Onset and alert \| Impact and management \| Recovery and learning \| \| --- \| --- \| --- \| --- \| --- \| \| Governance \|  \|  \|  \|  \| \| Resource generation \|  \|  \|  \|  \| \| Financing \|  \|  \|  \|  \| \| Service delivery \|  \|  \|  \|  \|   Q2. Which dimensions of the health system did the discussion cover?   \| Area \| Sub-area / definition \| 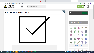covered \| \| --- \| --- \| --- \| \| WHO HSPA framework \| \| \| \| Governance \| Policy and division \|  \| \| Stakeholder voice \|  \| \| Information and intelligence \|  \| \| Legislation and regulation \|  \| \| Resource generation \| Health workforce \|  \| \| Infrastructure and medical equipment \|  \| \| Pharmaceuticals and other consumables \|  \| \| Financing \| Revenue raising \|  \| \| Pooling \|  \| \| Purchasing \|  \| \| Service delivery \| Public health \|  \| \| Primary care \|  \| \| Specialist care \|  \| \| Outcomes of health care system \| \| \| \| Effectiveness \| achieving desirable outcomes, given the correct provision of services to all who could benefit \|  \| \| Safety \| avoiding, preventing, and ameliorating adverse outcomes that stem from the processes of health care itself \|  \| \| People centeredness \| Placing the patients/carers at the center of care delivery \|  \| \| Efficiency \| Optimal use of available resources to yield maximum benefits \|  \| \| Equity \| (Ensuring distribution of care quality and access that does not vary because of personal characteristics \|  \| |
| Q3. In the discussion of this pilot, how is each function interconnected to other functions across different shock stages? Connect the first- and second order effects across functions and stages with lines and arrow.  (e.g., Resource generation in preparedness → Service delivery in Impact and management)   \|  \| Preparedness \| Onset and alert \| Impact and management \| Recovery and learning \| \| --- \| --- \| --- \| --- \| --- \| \| Governance \|  \|  \|  \|  \| \| Resource generation \|  \|  \|  \|  \| \| Financing \|  \|  \|  \|  \| \| Service delivery \|  \|  \|  \|  \| |
| Q4. What topics did the discussion cover?   \| Topics the scenario intended to introduce \| deemed important /  deemed unimportant \| Why? \| \| --- \| --- \| --- \| \| *Depending on the scenario*  *e.g., Finland: Protection of children, impacts of the recent reform* \|  \|  \| \|  \|  \|  \| \|  \|  \|  \|  \| Topics the scenario did not intend to introduce  (non-scenario specific) \| deemed important /  deemed unimportant \| Why? \| \| --- \| --- \| --- \| \|  \|  \|  \| \|  \|  \|  \| \|  \|  \|  \|   Further comments |
| 1. **Discussion flow and methodology** |
| Q1. Was there sufficient time to run through the whole exercise?   \|  \|  \| \| --- \| --- \| \| Yes \| No \|   Q2. From the perspective of observers, did the discussion flow without significant disruptions? (e.g., unmotivated participants, distractions, confusion of scenario, unnecessary breaks)   \|  \|  \|  \|  \|  \| \| --- \| --- \| --- \| --- \| --- \| \| 1 Not at all \| 2 \| 3 \| 4 \| 5 Very well \|   Q3. If a facilitation methodology was used (e.g., Finland “Me-We-Us” method), did it contribute to improving the discussion?   \|  \|  \|  \|  \|  \| \| --- \| --- \| --- \| --- \| --- \| \| 1 Not at all \| 2 \| 3 \| 4 \| 5 Very well \|     Q4. Did the discussion go beyond the scenario to discuss the health care system in general or non-scenario specific resilience?   \|  \|  \|  \|  \|  \| \| --- \| --- \| --- \| --- \| --- \| \| 1 Not at all \| 2 \| 3 \| 4 \| 5 Very well \|   Q5. Did the visual tools serve as good presentation tools to help the facilitator and participants to proceed with the discussion?   \|  \|  \|  \|  \|  \| \| --- \| --- \| --- \| --- \| --- \| \| 1 Not at all \| 2 \| 3 \| 4 \| 5 Very well \|   Q6. Did the participants build a consensus on the results of the discussion?   \|  \|  \|  \|  \|  \| \| --- \| --- \| --- \| --- \| --- \| \| 1 Not at all \| 2 \| 3 \| 4 \| 5 Very well \|   Further comments  (e.g., What did go well and what not? What elements should be retained/changed for future pilots?) |
| 1. **Description and involvement of the participants** |
| Q1. From the perspective of observers, do the participants represent different actors of the country’s health system?   \|  \|  \|  \|  \|  \| \| --- \| --- \| --- \| --- \| --- \| \| 1 Not at all \| 2 \| 3 \| 4 \| 5 Very well \|   Q2. What is the affiliation of participants?   \| Affiliation of participants \| 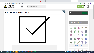 \| \| --- \| --- \| \| Line ministries / departments \|  \| \| Health care providers  (Health professionals, health facilities, associations) \|  \| \| Academic / research organisations \|  \| \| Civil society organisations /  Non-profit organisations \|  \| \| Private sector \|  \| \| Others \|  \|   Q3. Were participants well-informed about the scenario?   \|  \|  \|  \|  \|  \| \| --- \| --- \| --- \| --- \| --- \| \| 1 Not at all \| 2 \| 3 \| 4 \| 5 Very well \|   Q4. Did the participants take the pilot seriously?   \|  \|  \|  \|  \|  \| \| --- \| --- \| --- \| --- \| --- \| \| 1 Not at all \| 2 \| 3 \| 4 \| 5 Very well \|   Q5. Did the participants have sufficient knowledge of their health system to contribute to the discussion?   \|  \|  \|  \|  \|  \| \| --- \| --- \| --- \| --- \| --- \| \| 1 Not at all \| 2 \| 3 \| 4 \| 5 Very well \|   Q6. Did most of the participants actively engage in the discussion by providing sufficient input based on their area of expertise and responding to input given by other participants?   \|  \|  \|  \|  \|  \| \| --- \| --- \| --- \| --- \| --- \| \| 1 Not at all \| 2 \| 3 \| 4 \| 5 Very well \|   Q7. From the perspective of observers, did the participants cooperate/constructively interact with each other to feed the discussion?   \|  \|  \|  \|  \|  \| \| --- \| --- \| --- \| --- \| --- \| \| 1 Not at all \| 2 \| 3 \| 4 \| 5 Very well \|   Q8. How reasonable were the justifications for arguments made by the participants?   \|  \|  \|  \|  \|  \| \| --- \| --- \| --- \| --- \| --- \| \| 1 Not at all \| 2 \| 3 \| 4 \| 5 Very well \|   Further comments  (e.g., what action can participants do better or differently? What action contributed to the discussion? What was missing? |
| 1. **Role of the facilitator** |
| Q1. Did the facilitator clarify the objectives of the pilot?   \|  \|  \|  \|  \|  \| \| --- \| --- \| --- \| --- \| --- \| \| 1 Not at all \| 2 \| 3 \| 4 \| 5 Very well \|   Q2. Did the facilitator ensure that all the required processes were adhered?   \|  \|  \|  \|  \|  \| \| --- \| --- \| --- \| --- \| --- \| \| 1 Not at all \| 2 \| 3 \| 4 \| 5 Very well \|   Q3. Did the facilitator provide sufficient information to the participants?   \|  \|  \|  \|  \|  \| \| --- \| --- \| --- \| --- \| --- \| \| 1 Not at all \| 2 \| 3 \| 4 \| 5 Very well \|   Q4. Did the facilitator stay objective and neutral?   \|  \|  \|  \|  \|  \| \| --- \| --- \| --- \| --- \| --- \| \| 1 Not at all \| 2 \| 3 \| 4 \| 5 Very well \|   Q5. Did the facilitator encourage cooperation and interaction among the participants?   \|  \|  \|  \|  \|  \| \| --- \| --- \| --- \| --- \| --- \| \| 1 Not at all \| 2 \| 3 \| 4 \| 5 Very well \|   Q6. (If applicable) Was the facilitator able to handle tensions among the participants?   \|  \|  \|  \|  \|  \| \| --- \| --- \| --- \| --- \| --- \| \| 1 Not at all \| 2 \| 3 \| 4 \| 5 Very well \|   Q7. (If applicable) Was the facilitator able to handle tensions or conflict of interest situations?   \|  \|  \|  \|  \|  \| \| --- \| --- \| --- \| --- \| --- \| \| 1 Not at all \| 2 \| 3 \| 4 \| 5 Very well \|   Q8. Did the facilitator involve every participant by encouraging less active participants to engage in the discussion?   \|  \|  \|  \|  \|  \| \| --- \| --- \| --- \| --- \| --- \| \| 1 Not at all \| 2 \| 3 \| 4 \| 5 Very well \|   Further comments  (e.g., how was the overall impression of the facilitator? What action can facilitators do better or differently? What action contributed to the discussion? What was missing? What went well? What facilitation style was used?) |
|  |
| 1. **Overall evaluation** |
| Q1. How do you assess how the pilot went overall?   \|  \|  \|  \|  \|  \| \| --- \| --- \| --- \| --- \| --- \| \| 1 Poor \| 2 \| 3 \| 4 \| 5 Excellent \|   Further comments  (e.g., What went well?, what are the limitations?) |

## Part 2. Assessment of the pilot results

The second part of this assessment template intends to evaluate the outcomes of the pilot, focusing on the test country’s health system resilience in the context of a shock scenario. It contains 12 questions divided into 2 sections:

1. Pilot results
2. Test country’s health system resilience

| 1. **Pilot results** |
| --- |
| Q1. Did the discussion identify vulnerabilities within the country’s health system that the scenario intended to introduce?   \|  \|  \| \| --- \| --- \| \| Yes \| No \|   Q2. Did the discussion identify unintended or previously unnoticed vulnerabilities or strengths?   \|  \|  \| \| --- \| --- \| \| Yes \| No \|   Q3. If “Yes” for Q1 or/and Q2, what potential consequences and countermeasures were discussed?   \| Strengths \| Vulnerabilities \| Consequences  (if applicable) \| Countermeasures  (if applicable) \| \| --- \| --- \| --- \| --- \| \|  \|  \|  \|  \| \|  \|  \|  \|  \| \|  \|  \|  \|  \| \|  \|  \|  \|  \|   Q4. Should a shock similar to the one described in the scenario occur, would the measures drawn from the discussion be effective in addressing vulnerabilities?   \|  \|  \|  \|  \|  \| \| --- \| --- \| --- \| --- \| --- \| \| 1 Not at all \| 2 \| 3 \| 4 \| 5 Very well \|   Q5. Are the measures drawn from the discussion applicable/implementable in reality?   \|  \|  \|  \|  \|  \|  \| \| --- \| --- \| --- \| --- \| --- \| --- \| \| Do not know \| 1 Not at all \| 2 \| 3 \| 4 \| 5 Very well \|   Q6. Are there vulnerabilities that the discussion overlooked?   \|  \|  \| \| --- \| --- \| \| Yes \| No \|   Q7. If the answers to Q6 is yes, what are they and why were they overlooked? What might it imply about the country’s health care system?  Further comments  (e.g., validity and reliability of the results) |
| 1. **Test country’s health system resilience** |
| Q1. How resilient is the country’s health system in the framework of the HSPA functions? What are the strengths and weaknesses?   \| HSPA function \| HSPA subfunction \| 1  Poor \| 2 \| 3 \| 4 \| 5  Excellent \| \| --- \| --- \| --- \| --- \| --- \| --- \| --- \| \| Governance \| Policy and division \|  \|  \|  \|  \|  \| \| Stakeholder voice \|  \|  \|  \|  \|  \| \| Information and intelligence \|  \|  \|  \|  \|  \| \| Legislation and regulation \|  \|  \|  \|  \|  \| \| Resource generation \| Health workforce \|  \|  \|  \|  \|  \| \| Infrastructure and medical equipment \|  \|  \|  \|  \|  \| \| Pharmaceuticals and other consumables \|  \|  \|  \|  \|  \| \| Financing \| Revenue raising \|  \|  \|  \|  \|  \| \| Pooling \|  \|  \|  \|  \|  \| \| Purchasing \|  \|  \|  \|  \|  \| \| Service delivery \| Public health \|  \|  \|  \|  \|  \| \| Primary care \|  \|  \|  \|  \|  \| \| Specialist care \|  \|  \|  \|  \|  \|   Q2. How resilient would the HSPA functions be during each phase of the shock cycle?  (Not at all) 1 2 3 4 5 (Very well)   \|  \| Preparedness \| Onset and alert \| Impact and management \| Recovery and learning \| \| --- \| --- \| --- \| --- \| --- \| \| Governance \|  \|  \|  \|  \| \| Resource generation \|  \|  \|  \|  \| \| Financing \|  \|  \|  \|  \| \| Service delivery \|  \|  \|  \|  \|   Q3. What are the top strengths and weaknesses?   \| HSPA function \| S = strength  W = weakness \| HSPA subfunction \| S = strength  W = weakness \| \| --- \| --- \| --- \| --- \| \| Governance \|  \| Policy and division \|  \| \| Stakeholder voice \|  \| \| Information and intelligence \|  \| \| Legislation and regulation \|  \| \| Resource generation \|  \| Health workforce \|  \| \| Infrastructure and medical equipment \|  \| \| Pharmaceuticals and other consumables \|  \| \| Financing \|  \| Revenue raising \|  \| \| Pooling \|  \| \| Purchasing \|  \| \| Service delivery \|  \| Public health \|  \| \| Primary care \|  \| \| Specialist care \|  \|   Q4. What are the top 3 strengths and weaknesses, and what do they imply about the country’s health system?   \| Strengths \| Weaknesses \| Implications \| \| --- \| --- \| --- \| \|  \|  \|  \| \|  \|  \|  \| \|  \|  \|  \|   Q5. Can the country’s health system be viewed as resilient in the context of the scenario situation?   \|  \|  \|  \|  \|  \| \| --- \| --- \| --- \| --- \| --- \| \| 1 Not at all \| 2 \| 3 \| 4 \| 5 Very well \|   Q6. Can the country’s health system be viewed as resilient more generally speaking?   \|  \|  \|  \|  \|  \| \| --- \| --- \| --- \| --- \| --- \| \| 1 Not at all \| 2 \| 3 \| 4 \| 5 Very well \|   Further comments |

## Annex: Further comments

Discussion phases

| Introduction |
| --- |
|  |
| Discussion |
|  |
| Conclusion |
|  |

Shock cycle

1. Preparedness
2. Onset & alert
3. Impact & management
4. Recovery and learning

# Questionnaire results


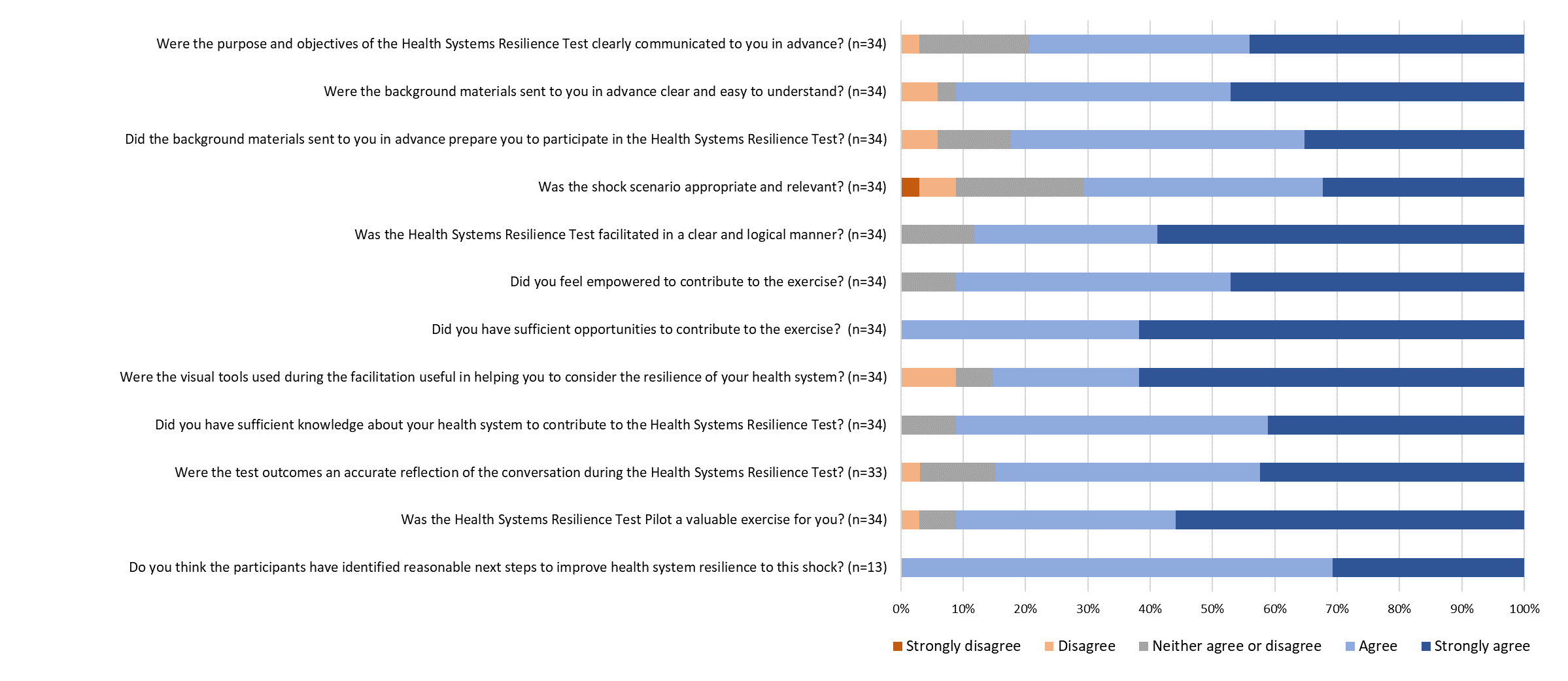


Supplementary material figure 1. Responses to Likert style questions

Supplementary material Table 1. Responses to yes-no style questions

|  | *n* | *n* responses Yes (%) | *n* responses No (%) |
| --- | --- | --- | --- |
| Did you have sufficient time to run through the exercise and consider the resilience of your health system in response to the shock scenario? | 34 | 33 (97%) | 1 (3%) |
| Were the results of the day consistent with your expectations of the major weaknesses of the health system? | 33 | 31 (94%) | 2 (6%) |
| Do you think that findings from the Health Systems Resilience Test will help you to identify and understand broader vulnerabilities in your health system (not specific to the shock scenario)? | 34 | 33 (97%) | 1 (3%) |
| Do you think you or the participants as a group might identify different strengths and weaknesses in the resilience of your health system if you tested it with a different shock scenario? | 34 | 26 (76%) | 8 (24%) |
